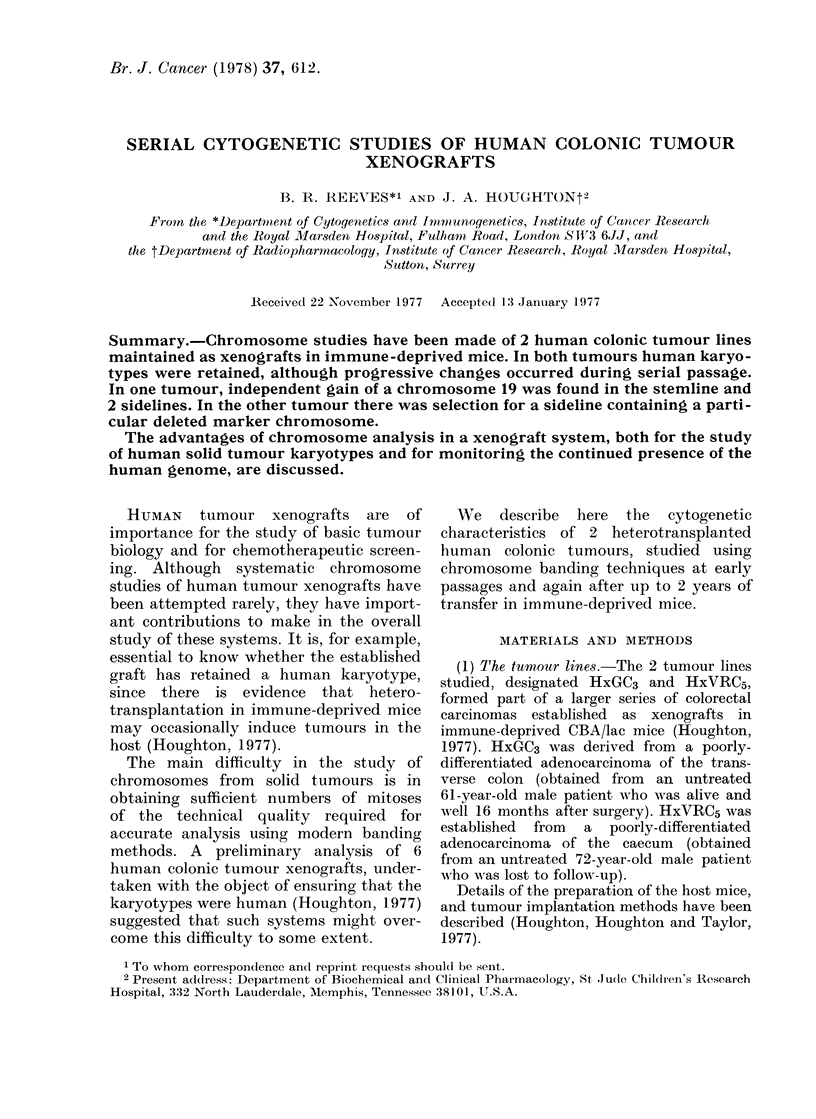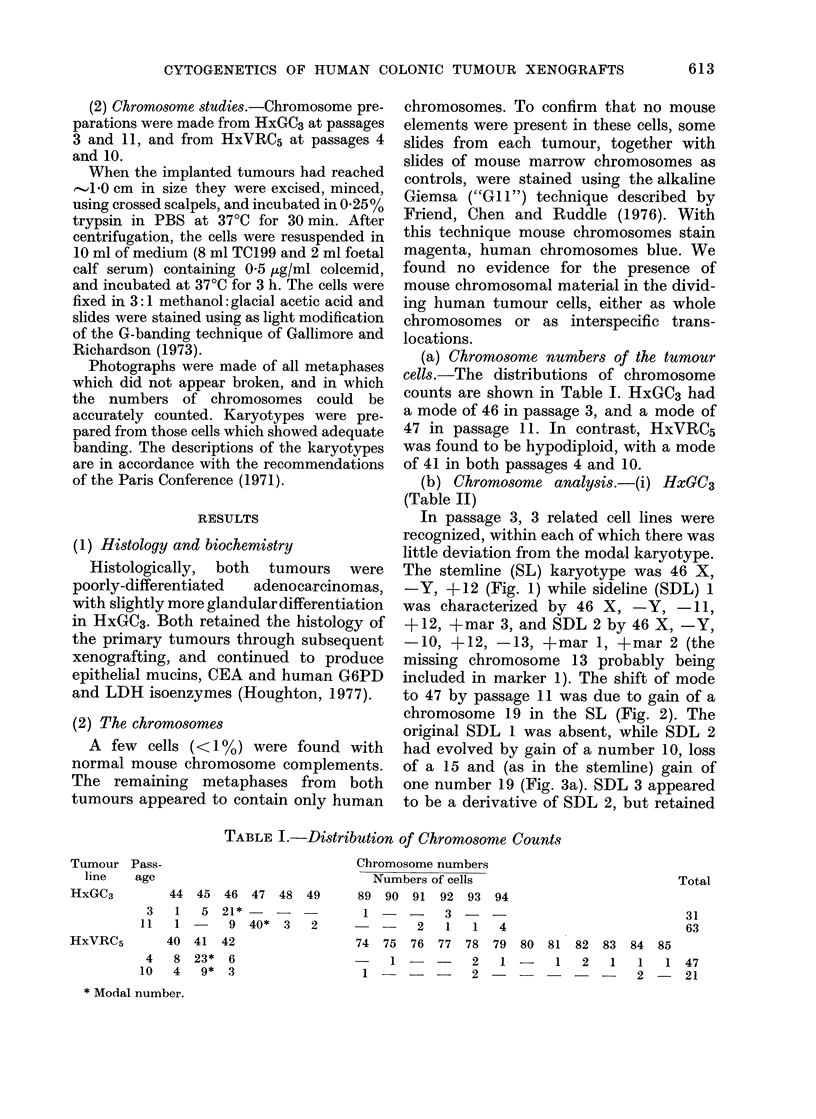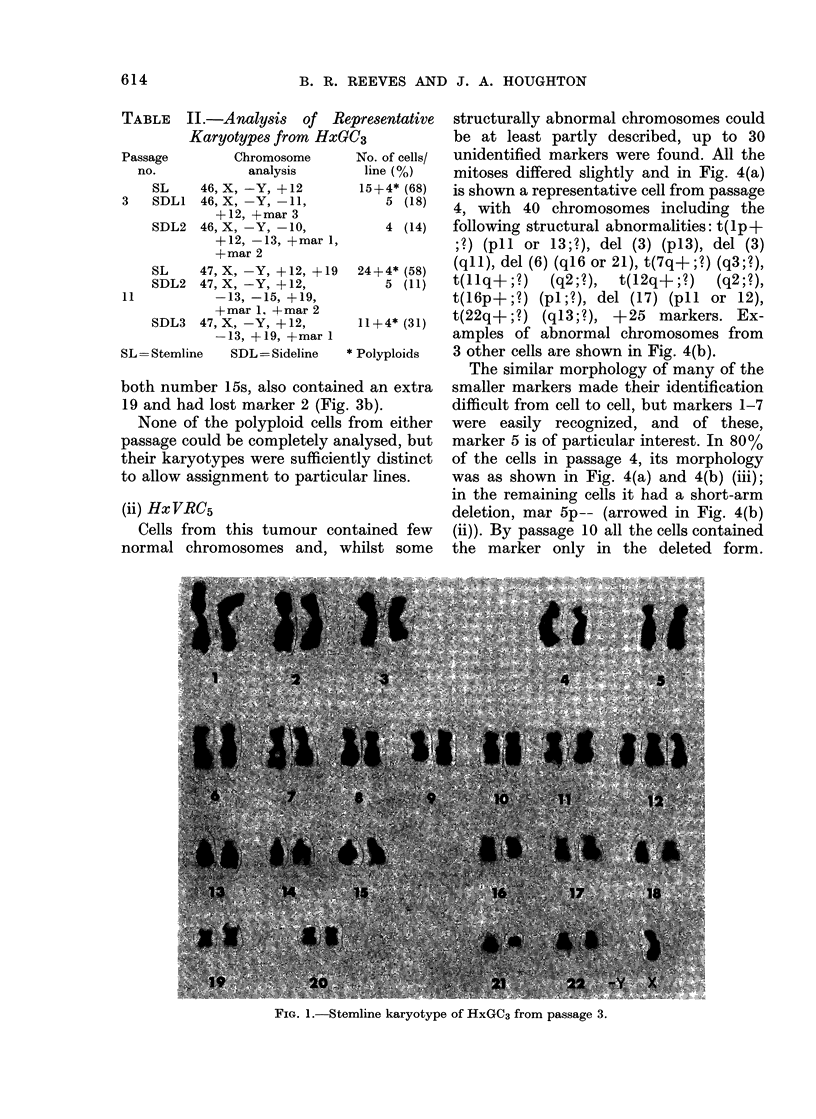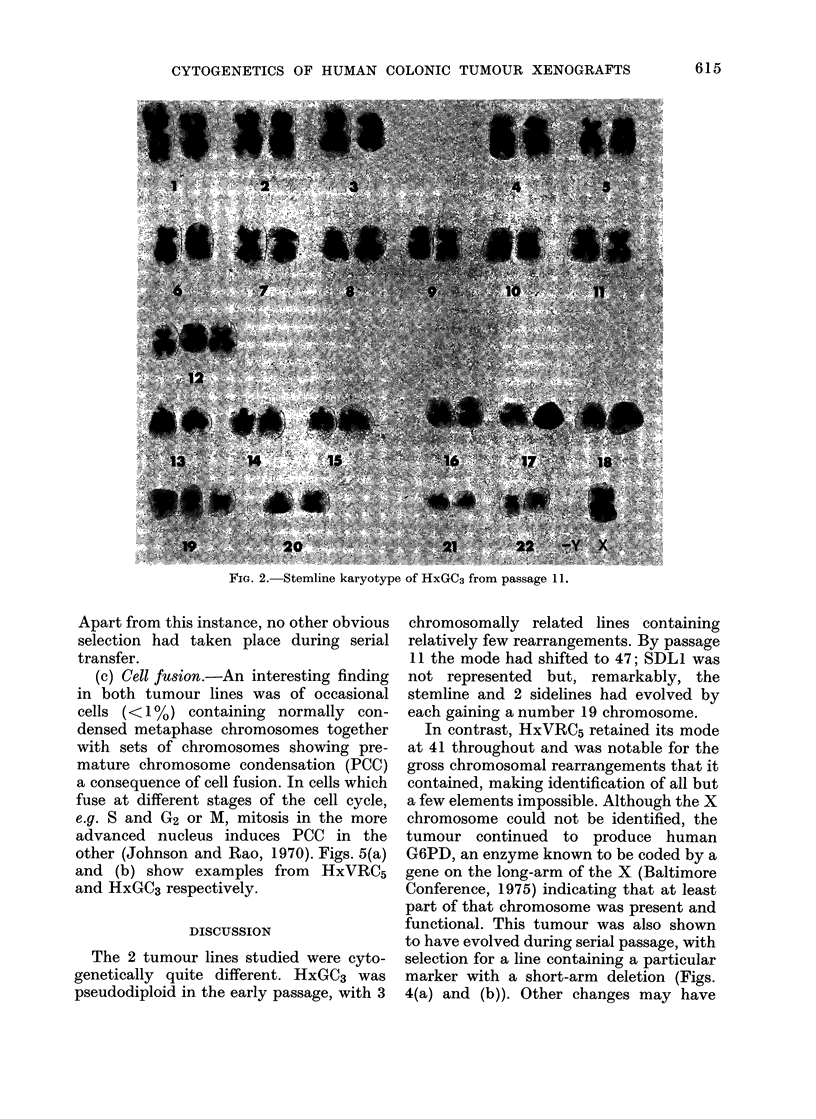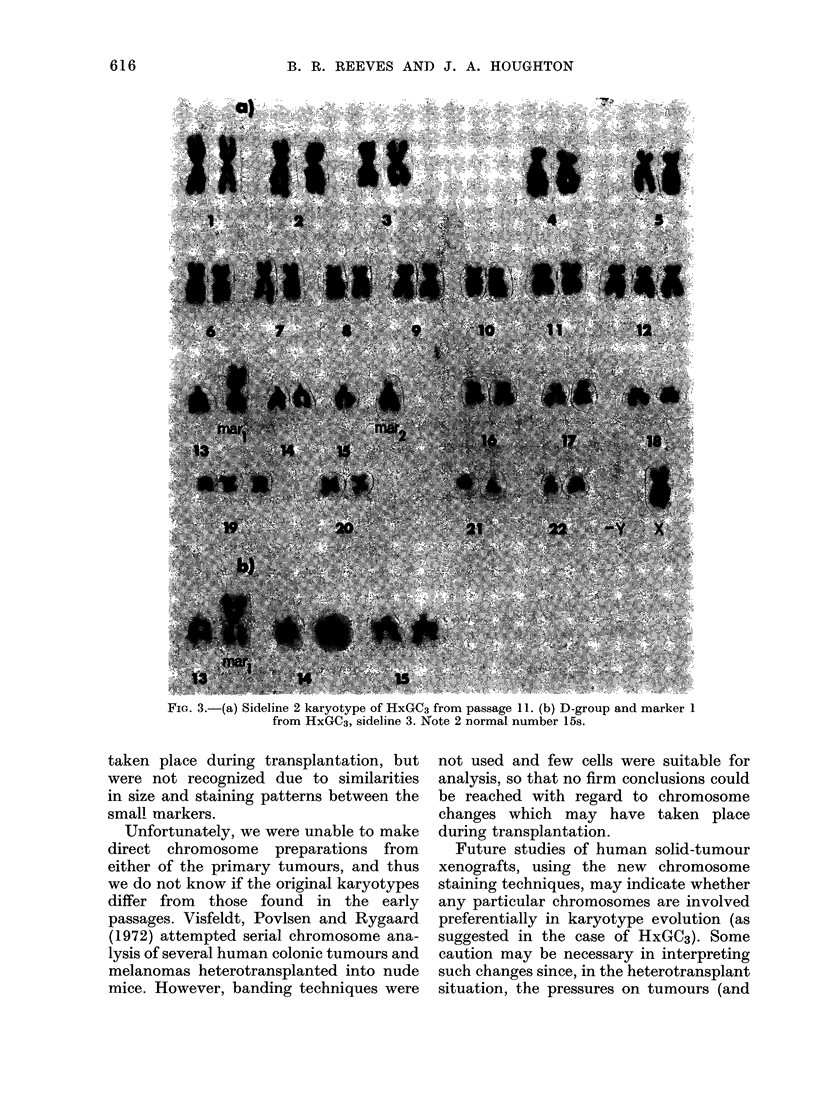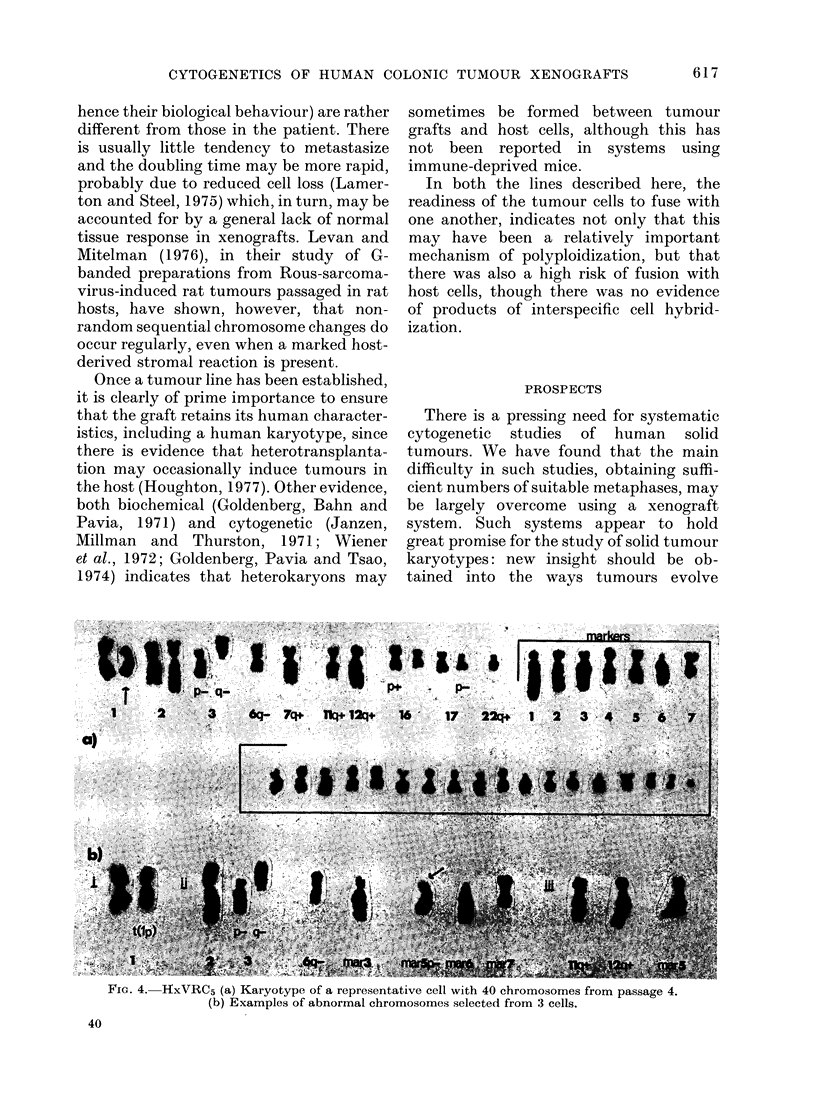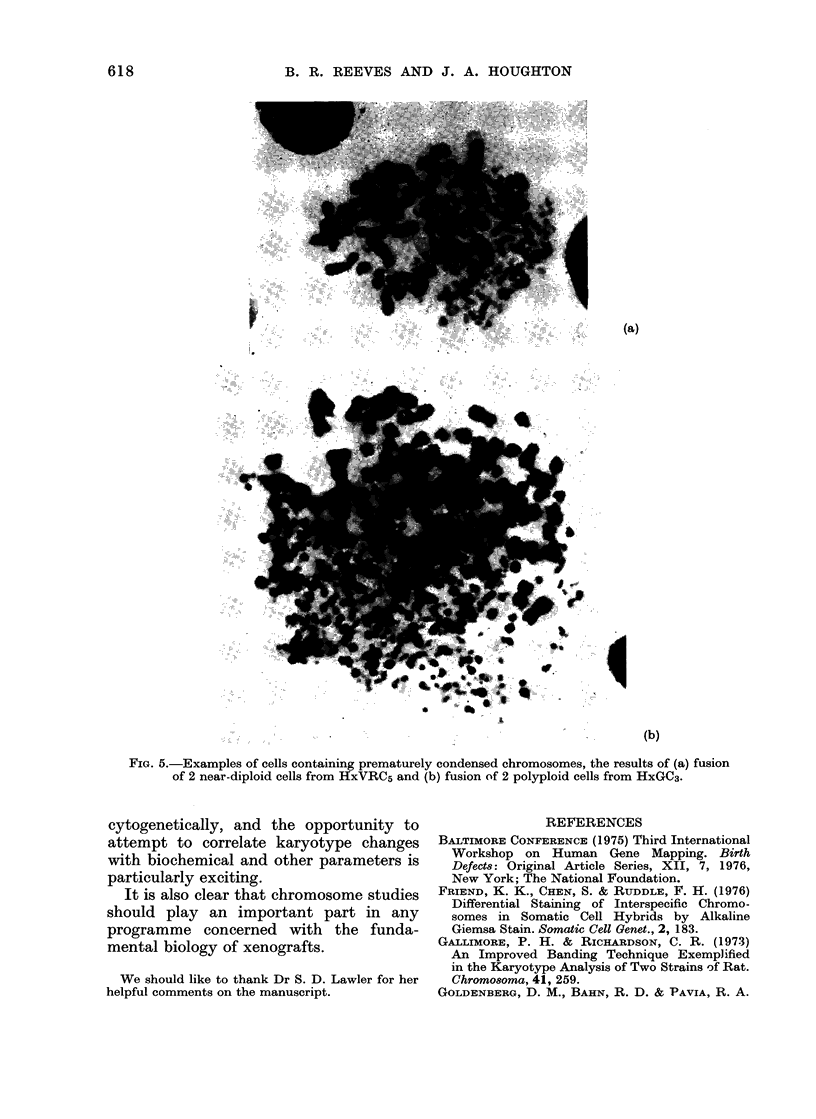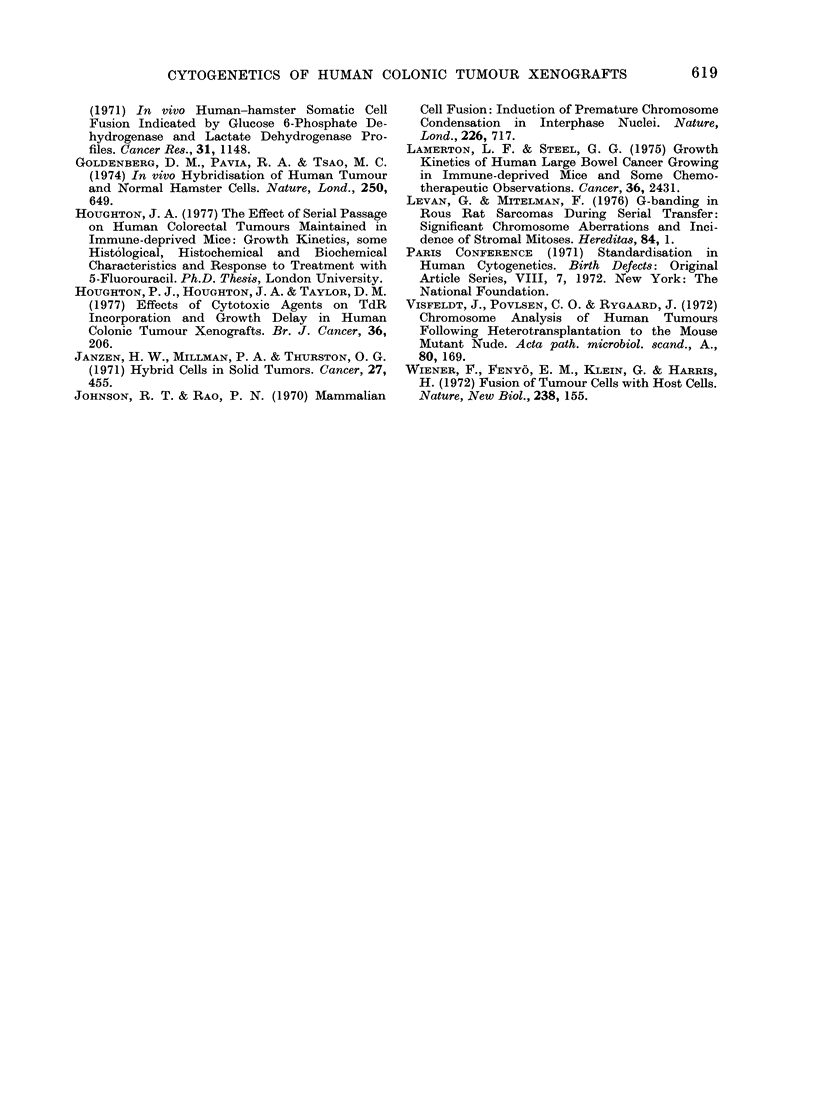# Serial cytogenetic studies of human colonic tumour xenografts.

**DOI:** 10.1038/bjc.1978.91

**Published:** 1978-04

**Authors:** B. R. Reeves, J. A. Houghton

## Abstract

**Images:**


Br. J. Cancer (1978) 37, 612.

### SERIAL CYTOGENETIC STUDIES OF HUMAN COLONIC TUMOUR XENOGRAFTS

B. R. REEVES*[1] AND J. A. HOUGHTON†[2]

*From the *Department of Cytogenetics and Immunogenetics, Institute of Cancer Research and the Royal Marsden Hospital, Fulham Road, London SW3 6JJ, and the †Department of Radiopharmacology, Institute of Cancer Research, Royal Marsden Hospital, Sutton, Surrey*

Received 22 November 1977 Accepted 13 January 1977

**Summary.—Chromosome studies have been made of 2 human colonic tumour lines maintained as xenografts in immune-deprived mice. In both tumours human karyotypes were retained, although progressive changes occurred during serial passage. In one tumour, independent gain of a chromosome 19 was found in the stemline and 2 sidelines. In the other tumour there was selection for a sideline containing a particular deleted marker chromosome.**

**The advantages of chromosome analysis in a xenograft system, both for the study of human solid tumour karyotypes and for monitoring the continued presence of the human genome, are discussed.**

HUMAN tumour xenografts are of importance for the study of basic tumour biology and for chemotherapeutic screening. Although systematic chromosome studies of human tumour xenografts have been attempted rarely, they have important contributions to make in the overall study of these systems. It is, for example, essential to know whether the established graft has retained a human karyotype, since there is evidence that heterotransplantation in immune-deprived mice may occasionally induce tumours in the host (Houghton, 1977).

The main difficulty in the study of chromosomes from solid tumours is in obtaining sufficient numbers of mitoses of the technical quality required for accurate analysis using modern banding methods. A preliminary analysis of 6 human colonic tumour xenografts, undertaken with the object of ensuring that the karyotypes were human (Houghton, 1977) suggested that such systems might overcome this difficulty to some extent.

We describe here the cytogenetic characteristics of 2 heterotransplanted human colonic tumours, studied using chromosome banding techniques at early passages and again after up to 2 years of transfer in immune-deprived mice.

### MATERIALS AND METHODS

(1) *The tumour lines.*—The 2 tumour lines studied, designated $HxGC_3$ and $HxVRC_5$, formed part of a larger series of colorectal carcinomas established as xenografts in immune-deprived CBA/lac mice (Houghton, 1977). $HxGC_3$ was derived from a poorly-differentiated adenocarcinoma of the transverse colon (obtained from an untreated 61-year-old male patient who was alive and well 16 months after surgery). $HxVRC_5$ was established from a poorly-differentiated adenocarcinoma of the caecum (obtained from an untreated 72-year-old male patient who was lost to follow-up).

Details of the preparation of the host mice, and tumour implantation methods have been described (Houghton, Houghton and Taylor, 1977).

[1] To whom correspondence and reprint requests should be sent.

[2] Present address: Department of Biochemical and Clinical Pharmacology, St Jude Children's Research Hospital, 332 North Lauderdale, Memphis, Tennessee 38101, U.S.A.

(2) *Chromosome studies.*—Chromosome preparations were made from $HxGC_3$ at passages 3 and 11, and from $HxVRC_5$ at passages 4 and 10.

When the implanted tumours had reached ~1·0 cm in size they were excised, minced, using crossed scalpels, and incubated in 0·25% trypsin in PBS at 37°C for 30 min. After centrifugation, the cells were resuspended in 10 ml of medium (8 ml TC199 and 2 ml foetal calf serum) containing 0·5 $\mu$g/ml colcemid, and incubated at 37°C for 3 h. The cells were fixed in 3:1 methanol:glacial acetic acid and slides were stained using as light modification of the G-banding technique of Gallimore and Richardson (1973).

Photographs were made of all metaphases which did not appear broken, and in which the numbers of chromosomes could be accurately counted. Karyotypes were prepared from those cells which showed adequate banding. The descriptions of the karyotypes are in accordance with the recommendations of the Paris Conference (1971).

### RESULTS

### (1) *Histology and biochemistry*

Histologically, both tumours were poorly-differentiated adenocarcinomas, with slightly more glandular differentiation in $HxGC_3$. Both retained the histology of the primary tumours through subsequent xenografting, and continued to produce epithelial mucins, CEA and human G6PD and LDH isoenzymes (Houghton, 1977).

### (2) *The chromosomes*

A few cells (<1%) were found with normal mouse chromosome complements. The remaining metaphases from both tumours appeared to contain only human chromosomes. To confirm that no mouse elements were present in these cells, some slides from each tumour, together with slides of mouse marrow chromosomes as controls, were stained using the alkaline Giemsa ("G11") technique described by Friend, Chen and Ruddle (1976). With this technique mouse chromosomes stain magenta, human chromosomes blue. We found no evidence for the presence of mouse chromosomal material in the dividing human tumour cells, either as whole chromosomes or as interspecific translocations.

(a) *Chromosome numbers of the tumour cells.*—The distributions of chromosome counts are shown in Table I. $HxGC_3$ had a mode of 46 in passage 3, and a mode of 47 in passage 11. In contrast, $HxVRC_5$ was found to be hypodiploid, with a mode of 41 in both passages 4 and 10.

(b) *Chromosome analysis.*—(i) *$HxGC_3$* (Table II)

In passage 3, 3 related cell lines were recognized, within each of which there was little deviation from the modal karyotype. The stemline (SL) karyotype was 46 X, −Y, +12 (Fig. 1) while sideline (SDL) 1 was characterized by 46 X, −Y, −11, +12, +mar 3, and SDL 2 by 46 X, −Y, −10, +12, −13, +mar 1, +mar 2 (the missing chromosome 13 probably being included in marker 1). The shift of mode to 47 by passage 11 was due to gain of a chromosome 19 in the SL (Fig. 2). The original SDL 1 was absent, while SDL 2 had evolved by gain of a number 10, loss of a 15 and (as in the stemline) gain of one number 19 (Fig. 3a). SDL 3 appeared to be a derivative of SDL 2, but retained

TABLE I.—*Distribution of Chromosome Counts*

| Tumour line | Passage | Chromosome numbers / Numbers of cells | | | | | | | | | | | | | | | | | | Total |
|---|---|---|---|---|---|---|---|---|---|---|---|---|---|---|---|---|---|---|---|---|
| $HxGC_3$ | | 44 | 45 | 46 | 47 | 48 | 49 | 89 | 90 | 91 | 92 | 93 | 94 | | | | | | | |
| | 3 | 1 | 5 | 21* | — | — | — | 1 | — | — | 3 | — | — | | | | | | | 31 |
| | 11 | 1 | — | 9 | 40* | 3 | 2 | — | — | 2 | 1 | 1 | 4 | | | | | | | 63 |
| $HxVRC_5$ | | 40 | 41 | 42 | | | | 74 | 75 | 76 | 77 | 78 | 79 | 80 | 81 | 82 | 83 | 84 | 85 | |
| | 4 | 8 | 23* | 6 | | | | — | 1 | — | — | 2 | 1 | — | 1 | 2 | 1 | 1 | 1 | 47 |
| | 10 | 4 | 9* | 3 | | | | 1 | — | — | — | 2 | — | — | — | — | — | 2 | — | 21 |

* Modal number.

TABLE II.—*Analysis of Representative Karyotypes from HxGC*$_3$

| Passage no. | | Chromosome analysis | No. of cells/ line (%) |
|---|---|---|---|
| | SL | 46, X, −Y, +12 | 15+4* (68) |
| 3 | SDL1 | 46, X, −Y, −11, +12, +mar 3 | 5 (18) |
| | SDL2 | 46, X, −Y, −10, +12, −13, +mar 1, +mar 2 | 4 (14) |
| | SL | 47, X, −Y, +12, +19 | 24+4* (58) |
| 11 | SDL2 | 47, X, −Y, +12, −13, −15, +19, +mar 1, +mar 2 | 5 (11) |
| | SDL3 | 47, X, −Y, +12, −13, +19, +mar 1 | 11+4* (31) |

SL = Stemline SDL = Sideline * Polyploids both number 15s, also contained an extra 19 and had lost marker 2 (Fig. 3b).

None of the polyploid cells from either passage could be completely analysed, but their karyotypes were sufficiently distinct to allow assignment to particular lines.

(ii) *HxVRC*$_5$

Cells from this tumour contained few normal chromosomes and, whilst some structurally abnormal chromosomes could be at least partly described, up to 30 unidentified markers were found. All the mitoses differed slightly and in Fig. 4(a) is shown a representative cell from passage 4, with 40 chromosomes including the following structural abnormalities: t(1p+;?) (p11 or 13;?), del (3) (p13), del (3) (q11), del (6) (q16 or 21), t(7q+;?) (q3;?), t(11q+;?) (q2;?), t(12q+;?) (q2;?), t(16p+;?) (p1;?), del (17) (p11 or 12), t(22q+;?) (q13;?), +25 markers. Examples of abnormal chromosomes from 3 other cells are shown in Fig. 4(b).

The similar morphology of many of the smaller markers made their identification difficult from cell to cell, but markers 1–7 were easily recognized, and of these, marker 5 is of particular interest. In 80% of the cells in passage 4, its morphology was as shown in Fig. 4(a) and 4(b) (iii); in the remaining cells it had a short-arm deletion, mar 5p-- (arrowed in Fig. 4(b) (ii)). By passage 10 all the cells contained the marker only in the deleted form.

FIG. 1.—Stemline karyotype of HxGC$_3$ from passage 3.

FIG. 2.—Stemline karyotype of $HxGC_3$ from passage 11.

Apart from this instance, no other obvious selection had taken place during serial transfer.

(c) *Cell fusion.*—An interesting finding in both tumour lines was of occasional cells ($<1\%$) containing normally condensed metaphase chromosomes together with sets of chromosomes showing premature chromosome condensation (PCC) a consequence of cell fusion. In cells which fuse at different stages of the cell cycle, *e.g.* S and $G_2$ or M, mitosis in the more advanced nucleus induces PCC in the other (Johnson and Rao, 1970). Figs. 5(a) and (b) show examples from $HxVRC_5$ and $HxGC_3$ respectively.

### DISCUSSION

The 2 tumour lines studied were cytogenetically quite different. $HxGC_3$ was pseudodiploid in the early passage, with 3 chromosomally related lines containing relatively few rearrangements. By passage 11 the mode had shifted to 47; SDL1 was not represented but, remarkably, the stemline and 2 sidelines had evolved by each gaining a number 19 chromosome.

In contrast, $HxVRC_5$ retained its mode at 41 throughout and was notable for the gross chromosomal rearrangements that it contained, making identification of all but a few elements impossible. Although the X chromosome could not be identified, the tumour continued to produce human G6PD, an enzyme known to be coded by a gene on the long-arm of the X (Baltimore Conference, 1975) indicating that at least part of that chromosome was present and functional. This tumour was also shown to have evolved during serial passage, with selection for a line containing a particular marker with a short-arm deletion (Figs. 4(a) and (b)). Other changes may have

FIG. 3.—(a) Sideline 2 karyotype of $HxGC_3$ from passage 11. (b) D-group and marker 1 from $HxGC_3$, sideline 3. Note 2 normal number 15s.

taken place during transplantation, but were not recognized due to similarities in size and staining patterns between the small markers.

Unfortunately, we were unable to make direct chromosome preparations from either of the primary tumours, and thus we do not know if the original karyotypes differ from those found in the early passages. Visfeldt, Povlsen and Rygaard (1972) attempted serial chromosome analysis of several human colonic tumours and melanomas heterotransplanted into nude mice. However, banding techniques were not used and few cells were suitable for analysis, so that no firm conclusions could be reached with regard to chromosome changes which may have taken place during transplantation.

Future studies of human solid-tumour xenografts, using the new chromosome staining techniques, may indicate whether any particular chromosomes are involved preferentially in karyotype evolution (as suggested in the case of $HxGC_3$). Some caution may be necessary in interpreting such changes since, in the heterotransplant situation, the pressures on tumours (and hence their biological behaviour) are rather different from those in the patient. There is usually little tendency to metastasize and the doubling time may be more rapid, probably due to reduced cell loss (Lamerton and Steel, 1975) which, in turn, may be accounted for by a general lack of normal tissue response in xenografts. Levan and Mitelman (1976), in their study of G-banded preparations from Rous-sarcoma-virus-induced rat tumours passaged in rat hosts, have shown, however, that non-random sequential chromosome changes do occur regularly, even when a marked host-derived stromal reaction is present.

Once a tumour line has been established, it is clearly of prime importance to ensure that the graft retains its human characteristics, including a human karyotype, since there is evidence that heterotransplantation may occasionally induce tumours in the host (Houghton, 1977). Other evidence, both biochemical (Goldenberg, Bahn and Pavia, 1971) and cytogenetic (Janzen, Millman and Thurston, 1971; Wiener *et al.*, 1972; Goldenberg, Pavia and Tsao, 1974) indicates that heterokaryons may sometimes be formed between tumour grafts and host cells, although this has not been reported in systems using immune-deprived mice.

In both the lines described here, the readiness of the tumour cells to fuse with one another, indicates not only that this may have been a relatively important mechanism of polyploidization, but that there was also a high risk of fusion with host cells, though there was no evidence of products of interspecific cell hybridization.

### PROSPECTS

There is a pressing need for systematic cytogenetic studies of human solid tumours. We have found that the main difficulty in such studies, obtaining sufficient numbers of suitable metaphases, may be largely overcome using a xenograft system. Such systems appear to hold great promise for the study of solid tumour karyotypes: new insight should be obtained into the ways tumours evolve

FIG. 4.—HxVRC5 (a) Karyotype of a representative cell with 40 chromosomes from passage 4. (b) Examples of abnormal chromosomes selected from 3 cells.

(a)
(b)

FIG. 5.—Examples of cells containing prematurely condensed chromosomes, the results of (a) fusion of 2 near-diploid cells from $HxVRC_5$ and (b) fusion of 2 polyploid cells from $HxGC_3$.

cytogenetically, and the opportunity to attempt to correlate karyotype changes with biochemical and other parameters is particularly exciting.

It is also clear that chromosome studies should play an important part in any programme concerned with the fundamental biology of xenografts.

We should like to thank Dr S. D. Lawler for her helpful comments on the manuscript.

### REFERENCES

BALTIMORE CONFERENCE (1975) Third International Workshop on Human Gene Mapping. *Birth Defects*: Original Article Series, XII, 7, 1976, New York; The National Foundation.

FRIEND, K. K., CHEN, S. & RUDDLE, F. H. (1976) Differential Staining of Interspecific Chromosomes in Somatic Cell Hybrids by Alkaline Giemsa Stain. *Somatic Cell Genet.*, **2**, 183.

GALLIMORE, P. H. & RICHARDSON, C. R. (1973) An Improved Banding Technique Exemplified in the Karyotype Analysis of Two Strains of Rat. *Chromosoma*, **41**, 259.

GOLDENBERG, D. M., BAHN, R. D. & PAVIA, R. A.

(1971) *In vivo* Human–hamster Somatic Cell Fusion Indicated by Glucose 6-Phosphate Dehydrogenase and Lactate Dehydrogenase Profiles. *Cancer Res.*, **31**, 1148.

GOLDENBERG, D. M., PAVIA, R. A. & TSAO, M. C. (1974) *In vivo* Hybridisation of Human Tumour and Normal Hamster Cells. *Nature, Lond.*, **250**, 649.

HOUGHTON, J. A. (1977) The Effect of Serial Passage on Human Colorectal Tumours Maintained in Immune-deprived Mice: Growth Kinetics, some Histólogical, Histochemical and Biochemical Characteristics and Response to Treatment with 5-Fluorouracil. *Ph.D. Thesis*, London University.

HOUGHTON, P. J., HOUGHTON, J. A. & TAYLOR, D. M. (1977) Effects of Cytotoxic Agents on TdR Incorporation and Growth Delay in Human Colonic Tumour Xenografts. *Br. J. Cancer*, **36**, 206.

JANZEN, H. W., MILLMAN, P. A. & THURSTON, O. G. (1971) Hybrid Cells in Solid Tumors. *Cancer*, **27**, 455.

JOHNSON, R. T. & RAO, P. N. (1970) Mammalian Cell Fusion: Induction of Premature Chromosome Condensation in Interphase Nuclei. *Nature, Lond.*, **226**, 717.

LAMERTON, L. F. & STEEL, G. G. (1975) Growth Kinetics of Human Large Bowel Cancer Growing in Immune-deprived Mice and Some Chemotherapeutic Observations. *Cancer*, **36**, 2431.

LEVAN, G. & MITELMAN, F. (1976) G-banding in Rous Rat Sarcomas During Serial Transfer: Significant Chromosome Aberrations and Incidence of Stromal Mitoses. *Hereditas*, **84**, 1.

PARIS CONFERENCE (1971) Standardisation in Human Cytogenetics. *Birth Defects*: Original Article Series, VIII, 7, 1972. New York: The National Foundation.

VISFELDT, J., POVLSEN, C. O. & RYGAARD, J. (1972) Chromosome Analysis of Human Tumours Following Heterotransplantation to the Mouse Mutant Nude. *Acta path. microbiol. scand.*, A., **80**, 169.

WIENER, F., FENYÖ, E. M., KLEIN, G. & HARRIS, H. (1972) Fusion of Tumour Cells with Host Cells. *Nature, New Biol.*, **238**, 155.